# Robust nonparametric quantification of clustering density of molecules in single-molecule localization microscopy

**DOI:** 10.1371/journal.pone.0179975

**Published:** 2017-06-21

**Authors:** Shenghang Jiang, Seongjin Park, Sai Divya Challapalli, Jingyi Fei, Yong Wang

**Affiliations:** 1 Department of Physics, University of Arkansas, Fayetteville, Arkansas, 72701, United States of America; 2 Department of Biochemistry and Molecular Biology, The University of Chicago, Chicago, Illinois, 60637, United States of America; 3 Microelectronics and Photonics Graduate Program, University of Arkansas, Fayetteville, Arkansas, 72701, United States of America; 4 Institute of Biophysical Dynamics, The University of Chicago, Chicago, Illinois, 60637, United States of America; 5 Cell and Molecular Biology Program, University of Arkansas, Fayetteville, Arkansas, 72701, United States of America; J. Heyrovsky Institute of Physical Chemistry, CZECH REPUBLIC

## Abstract

We report a robust nonparametric descriptor, *J*′(*r*), for quantifying the density of clustering molecules in single-molecule localization microscopy. *J*′(*r*), based on nearest neighbor distribution functions, does not require any parameter as an input for analyzing point patterns. We show that *J*′(*r*) displays a valley shape in the presence of clusters of molecules, and the characteristics of the valley reliably report the clustering features in the data. Most importantly, the position of the *J*′(*r*) valley ($r_{J_{m}^{\prime }}$) depends exclusively on the density of clustering molecules (*ρ*_*c*_). Therefore, it is ideal for direct estimation of the clustering density of molecules in single-molecule localization microscopy. As an example, this descriptor was applied to estimate the clustering density of *ptsG* mRNA in *E. coli* bacteria.

## Introduction

Single-molecule localization microscopy (SMLM) has been utilized broadly in imaging biological molecules—proteins, DNA, and RNA—in various biological systems [1–5]. More importantly, by localizing individual molecules, SMLM has allowed quantitative analyses on the spatial organizations and patterns of these molecules, and produced new, quantitative and crucial information that was not accessible previously. New mechanisms of various cellular and molecular organizations and activities at the single-cell level have been unraveled using SMLM [6–15].

Many algorithms have been adopted, utilized, or developed, in the field of SMLM for analyzing localization data of molecules and quantifying inter-molecular organizations [13, 14, 16–23]. These methods provide means to identify statistically the forming of clustering molecules from random populations, to examine complex patterns of molecular organization, to segment molecules into clusters, and to quantify clustering features. For example, pair-correlation analysis has been applied to SMLM data on membrane proteins to identify the presence of clusters, as well as to estimate various cluster features, such as the density of molecules in a cluster and overall size of a cluster [16, 24, 25]. In addition, density-based algorithms such as DBSCAN (density-based spatial clustering of applications with noise) [26, 27] and OPTICS (ordering points to identify the clustering structure) [28, 29] have been exploited to identify clusters of proteins and nucleic acids, as well as to probe the clustering structures, in both bacteria and animal cells [13, 14, 17–19]. Other methods that have been used for analyzing SMLM data include Ripley’s *K*/*L*/*H* functions and their derivatives [20, 21]. More recently, Bayesian analysis and Voronoï diagrams have been utilized to segment molecules into clusters and to analyze the clustering properties [22, 23].

Segmentation and tessellation methods typically require human inputs as algorithm-parameters. For example, DBSCAN requires two parameters (a radius, eps, and the minimum number of points in the neighborhood for a point to be considered as a core point, minPts) [26–29], and they are known to be sensitive to the chosen parameters [18, 30]. The identification of clusters in the Voronoï diagram based method also requires a density threshold to determine whether points form clusters [23]. Although various techniques have been proposed to determine “appropriate” parameters for use [23, 27, 29, 31], bias is inevitably introduced by the choice of parameters in these algorithms.

It has been found that nonparametric algorithms could directly report some of the clustering features of molecules. For example, pair correlation analysis allowed to fit the computed correlation from experimental data to collect two fitting parameters that are coupled to the density of clustering points (*ρ*_*c*_), the number of clusters *N*_*c*_ and the density of random points *ρ*_*r*_ [16, 24, 25]. In addition, it has been reported that the derivative of Ripley’s *H* function, *H*′(*r*) gave the size of clusters (*R*_*c*_) reliably from the *r*-value corresponding to the minimum of *H*′(*r*): $r_{H_{m}^{\prime }}=2\times R_{c}$ [32, 33]. More importantly, it was found that $r_{H_{m}^{\prime }}$ only depends on the cluster size but insensitive to other clustering features such as the densities of clustering and random points [32].

Here we present another descriptor based on nearest neighbor distribution functions for directly reporting the density of clustering molecules (*ρ*_*c*_) in SMLM data. We examined the nearest neighbor function *G*(*r*) [34], the spherical contact distribution function *F*(*r*) [34], and the J-function *J*(*r*) = (1 − *G*(*r*)) / (1 − *F*(*r*)) [35, 36], and found that the associated derivative functions, *G*′(*r*) and *J*′(*r*), reliably report the clustering features of points. In the presence of clusters, *G*′(*r*) and *J*′(*r*) are peak/valley shaped. Most importantly, we observed that the position of the *J*′(*r*) valley, $r_{J_{m}^{\prime }}$, depends exclusively on the density of clustering points (*ρ*_*c*_). Therefore, unlike $r_{H_{m}^{\prime }}$ from Ripley’s *H* function that reports the cluster size, our descriptor, $r_{J_{m}^{\prime }}$, is ideal for direct measurements of the clustering density of molecules. As an example, we applied *J*′(*r*) and $r_{J_{m}^{\prime }}$ to estimate the clustering of *ptsG* mRNA in *E. coli*. We expect that this nonparametric descriptor, *J*′(*r*), together with *H*′(*r*) [32, 33], will be useful in a broad range of applications in SMLM.

## Results

### *G*(*r*), *F*(*r*) and *J*(*r*), and their derivatives

When quantifying the spatial organization of biological molecules in SMLM data, of particular interest in certain situations is the clustering or aggregation of molecules [37–40], which is featured by an enhancement in the local density of molecules. This enhancement in density has been used to identify clusters methods such as DBSCAN, OPTICS, and Voronoï tessellation [13, 14, 17–23]. On the other hand, the enhancement in the molecular density is also accompanied by the decrease of intermolecular distances, which could be described by functions based on nearest neighbor distances, such as pair-wise correlation function [16], nearest neighbor function *G*(*r*), and spherical contact distribution function *F*(*r*) [34]. The nearest neighbor function *G*(*r*) is the distribution function of the distance *r* of a point (existing in the data) to the nearest other point, while the spherical contact distribution *F*(*r*) is the distribution function of the distance *r* of an arbitrary point in the space (not necessarily existing in the data) to the nearest point in the data [34]. In addition, another function, *J*(*r*), has been suggested by van Lieshout and Baddeley in 1996 [35], $J\left(r\right)=\frac{1-G(r)}{1-F(r)}$, as a better nonparametric test to determine whether data were from a Poisson process.

**Fig 1:**
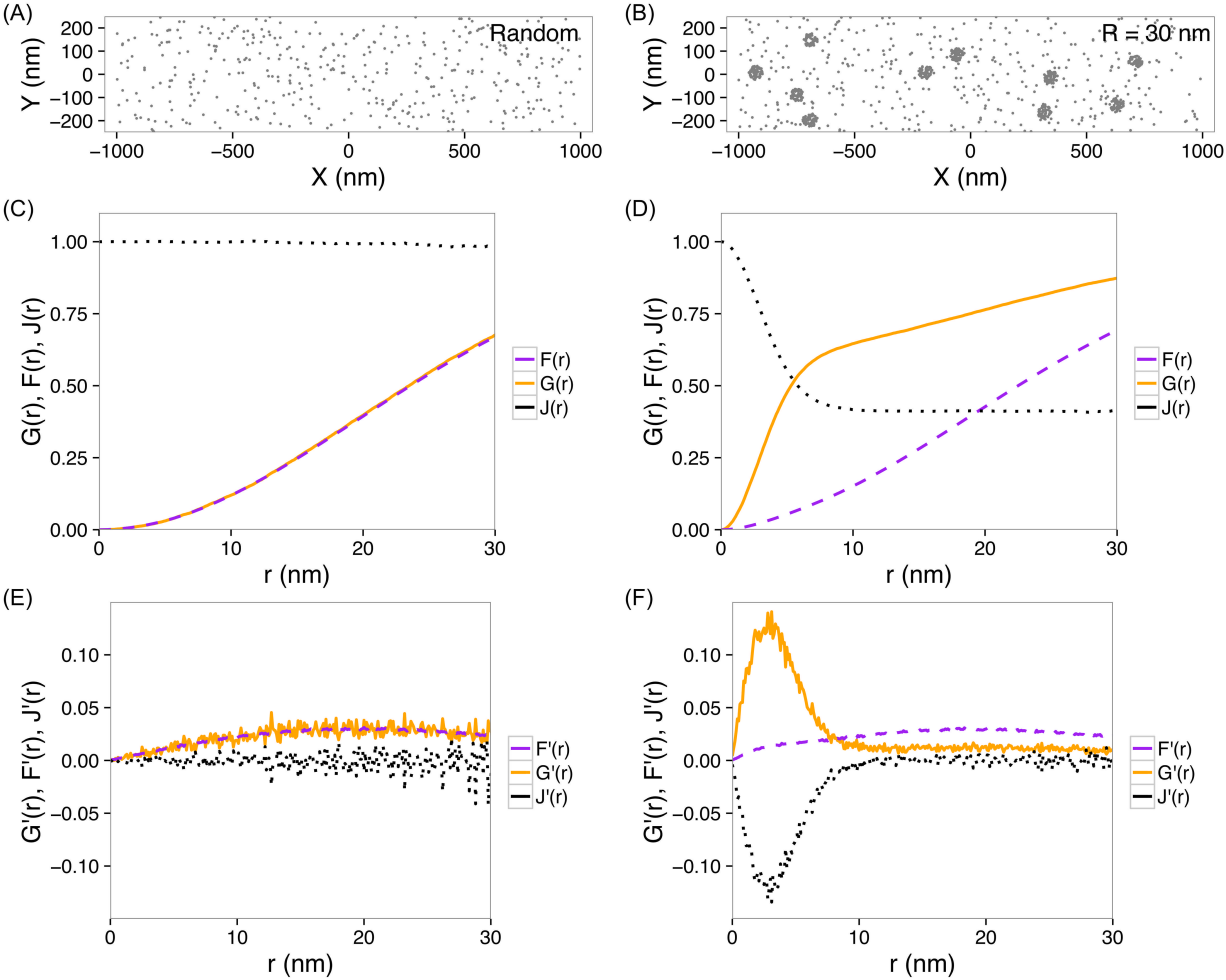
*G*(*r*), *F*(*r*) and *J*(*r*) functions, and their derivatives. (A) Simulated noise points. (B) Simulated points forming clusters with a radius of *R* = 30 nm, in the presence of noise points. (C, D) *G*(*r*), *F*(*r*) and *J*(*r*) functions calculated from the points in (A) and (B), respectively. (E, F) Derivatives, *G*′(*r*), *F*′(*r*) and *J*′(*r*), calculated from the points in (A) and (B), respectively.

We first explored how *G*(*r*), *F*(*r*) and *J*(*r*) functions depend on the clustering features of points using numerical simulations. Briefly, we generated points forming various clusters in the presence of noises (i.e., Poisson random points) in a region of interest, and computed these three functions. In a two-dimensional Poisson random process where points were not forming clusters (Fig 1A), the nearest neighbor functions gave the expected curves, *G*_*p*_(*r*) = *F*_*p*_(*r*) = 1 − exp(−λ*πr*^2^) (where λ is the density of points) and *J*_*p*_(*r*) = 1 (Fig 1C). However, when points aggregated into clusters (Fig 1B), both *G*(*r*) and *J*(*r*) deviated significantly from those for random points, while *F*(*r*) became only slightly different (Fig 1D). We observed that *J*(*r*) droped from 1 to ∼ 0.4 when *r* increased from 0 to 5 nm, while *G*(*r*) raised in the same *r*-range (0–5 nm). This observation indicates that *G*(*r*) and *J*(*r*) can be used for detection of clusters.

Furthermore, to remove accumulative effects, and inspired by Kiskowski et.al. [32], we calculated the derivatives of these functions: *G*′(*r*), *F*′(*r*) and *J*′(*r*). Striking peaks or valleys appeared in *G*′(*r*) and *J*′(*r*) if points formed clusters (Fig 1F). In contrast, these derivative functions remained essentially flat for random points (Fig 1E). On the other hand, *F*′(*r*)’s were very similar in the two cases (Fig 1E and 1F).

### Dependence of *G*′(*r*) and *J*′(*r*) functions on clustering features

**Fig 2:**
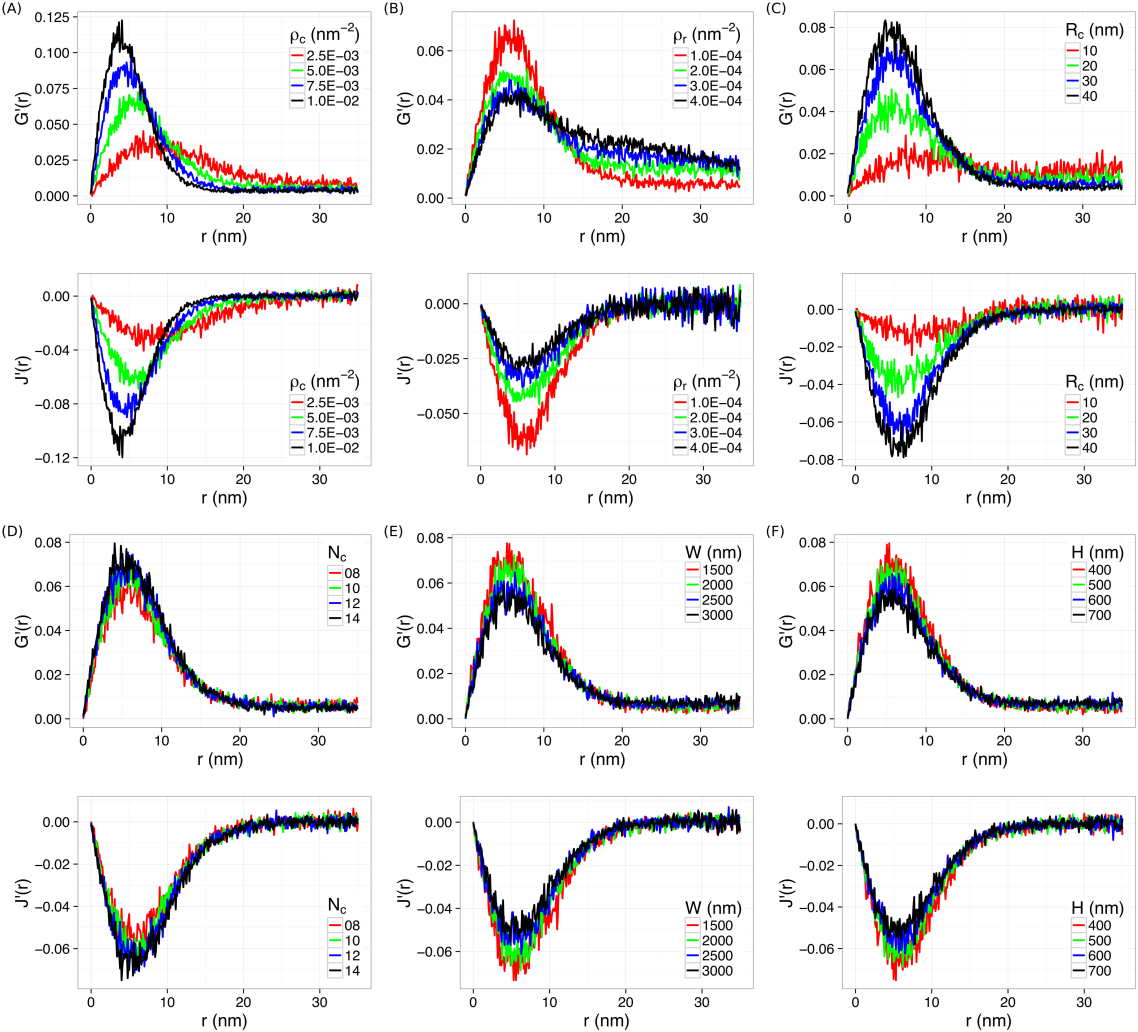
Changes in *G*′(*r*) and *J*′(*r*) by varying a cluster feature at a time. (A) *ρ*_*c*_, (B) *ρ*_*r*_, (C) *R*_*c*_, (D) *N*_*c*_, (E) *W*, and (F) *H*.

To explore quantitative applications of *G*′(*r*) and *J*′(*r*), we examined how they change with varying clustering features in the point patterns. Here we focused on the following features: the radius of clusters, *R*_*c*_, the density of clustering points (i.e., clustering density), *ρ*_*c*_, the number of clusters, *n*_*c*_, the density of random noise points (i.e., background points), *ρ*_*r*_, and the width (*W*) and height (*H*) of the region of interest (ROI). The first three features, *R*_*c*_, *ρ*_*c*_ and *n*_*c*_, are directly related to the properties of clusters in the data, while *ρ*_*r*_ is an indicator of the noise level. By varying one feature at a time, we observed that changes in *ρ*_*c*_, *ρ*_*r*_, or *R*_*c*_ resulted in horizontal shifting or vertical scaling of both *G*′(*r*) and *J*′(*r*) (Fig 2A–2C). For example, both *G*′(*r*) and *J*′(*r*) shifted to the left and scaled up when the clusters became denser (*ρ*_*c*_ increased). If the clusters became bigger (*R*_*c*_ increased) while keeping the clustering density constant, little horizontal translation was observed (Fig 2C), although both *G*′(*r*) and *J*′(*r*) scaled up too. In contrast, *G*′(*r*) and *J*′(*r*) were not as sensitive to the number of clusters (*N*_*c*_) or the size of the ROI, *W* and *H* (Fig 2D–2F).

**Fig 3:**
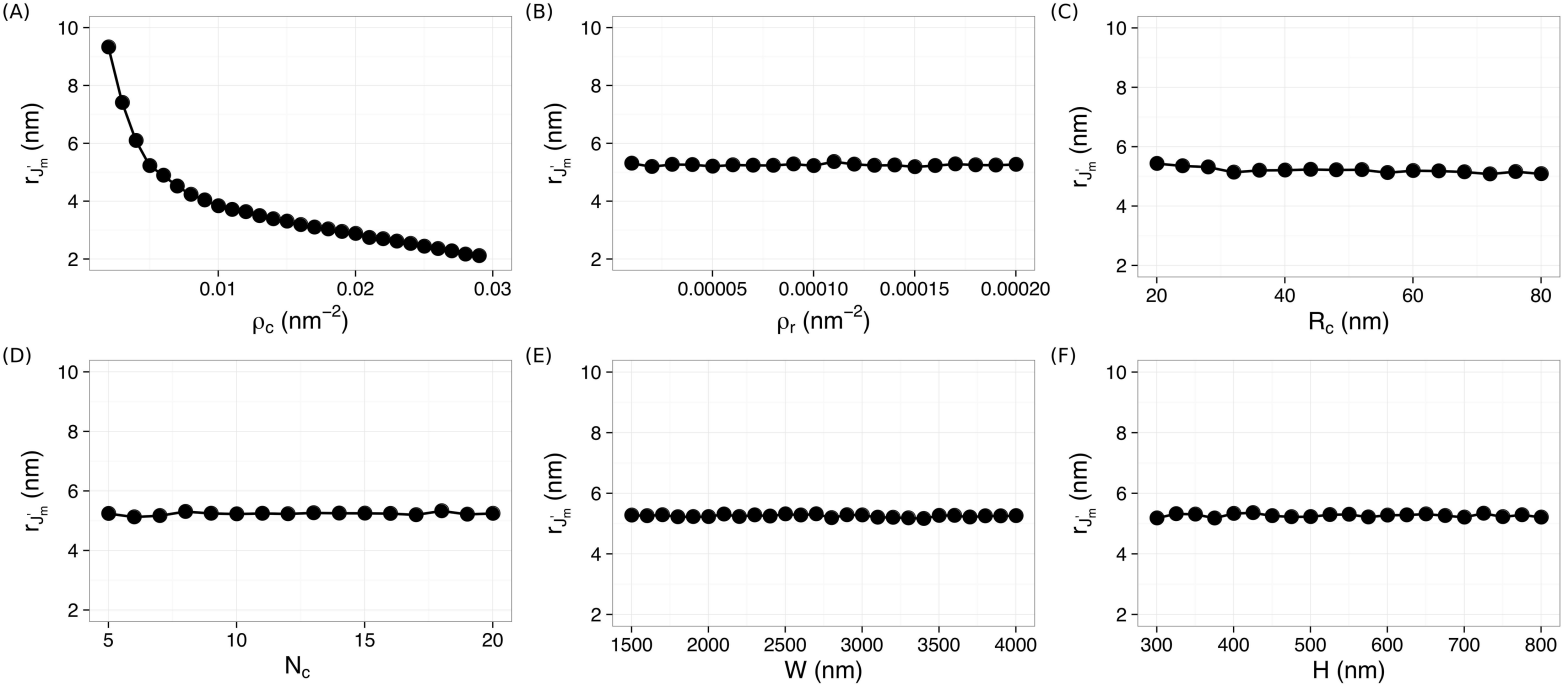
Dependence of $r_{J_{m}^{\prime }}$ on the clustering features. (A) *ρ*_*c*_, (B) *ρ*_*r*_, (C) *R*_*c*_, (D) *N*_*c*_, (E) *W*, and (F) *H*.

We further quantified the dependence of *G*′(*r*) and *J*′(*r*) on the clustering features. By fitting *G*′(*r*) and *J*′(*r*) with polynomials, both the amplitude (i.e., height of $G\prime (r):G_{m}^{\prime }$, or depth of $J\prime (r):J_{m}^{\prime }$) and the positions of the peaks and valleys ($r_{G_{m}^{\prime }}$ and $r_{J_{m}^{\prime }}$, respectively) were determined. The dependence of these values on the clustering features are shown in Fig 3, and S1–S3 Figs. We observed that both $G_{m}^{\prime }$ and $J_{m}^{\prime }$ depend on all the clustering features (S1 and S3 Figs), but $r_{G_{m}^{\prime }}$ and $r_{J_{m}^{\prime }}$ are most sensitive to the density of clustering points *ρ*_*c*_ (Fig 3 and S2 Fig). Most interestingly, $r_{J_{m}^{\prime }}$ is essentially independent on all the other clustering features except the density of clustering points *ρ*_*c*_ (Fig 3), providing a way to correlate $r_{J_{m}^{\prime }}$ with directly measuring the clustering densities of molecules, as shown below.

### Robust direct measurement of clustering density by $r_{J_{m}^{\prime }}$

**Fig 4:**
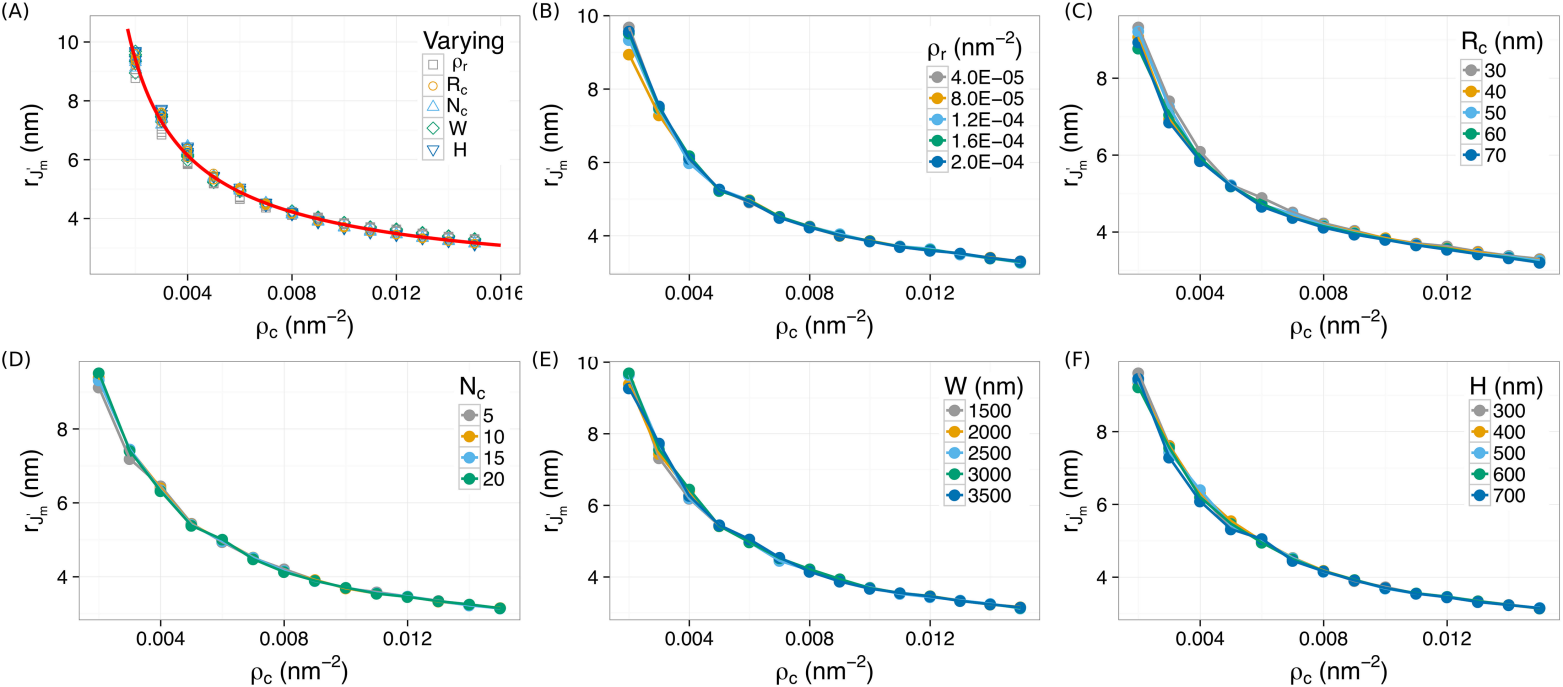
The $r_{J_{m}^{\prime }}\;-\;\rho_{c}$ relation is independent on all the other cluster features, *R*_*c*_, *ρ*_*r*_, *N*_*c*_, *W*, and *H*. All data points collapse onto a single power-law curve, $r_{J_{m}^{\prime }}=A\cdot {\rho_{c}}^{-\alpha }+b$. Least-square fitting gives *α* = 0.76 ± 0.03.

Our quantifier $r_{J_{m}^{\prime }}$ can be used for direct measurements of clustering densities of molecules. We first confirmed that the $r_{J_{m}^{\prime }}\;-\;\rho_{c}$ relation is independent on other clustering features when simultaneously varing both *ρ*_*c*_ and *R*_*c*_, or *N*_*c*_, or *ρ*_*r*_ ⋯. We found that the $r_{J_{m}^{\prime }}\;-\;\rho_{c}$ relation from all the simulations collapsed onto a single curve, as shown in Fig 4A. This curve was fitted very well (*R*^2^ = 0.9946) by a power-law function $r_{J_{m}^{\prime }}=A\cdot \rho_{c}^{-\alpha }+b$ with *α* = 0.76 ± 0.03. This curve provides a “calibration” that can be used to directly estimate the clustering density of molecules.

**Fig 5:**
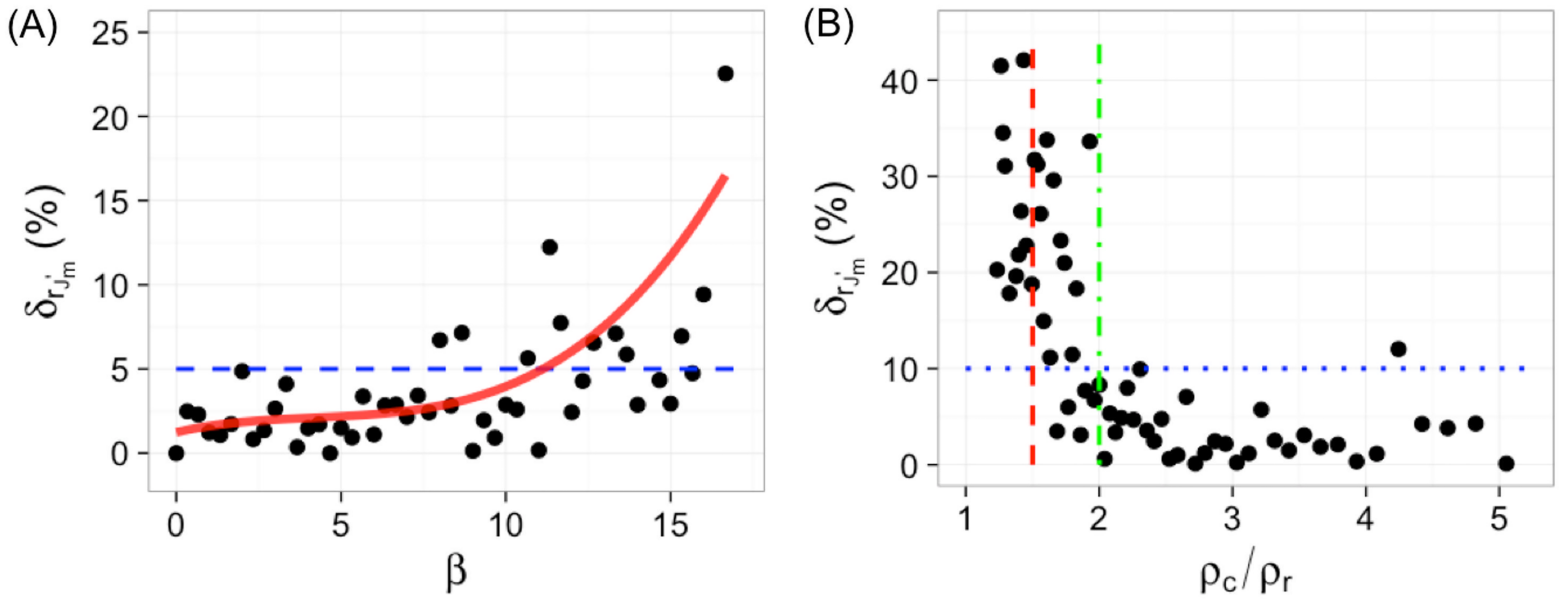
Robustness of the $r_{J_{m}^{\prime }}\;-\;\rho_{c}$ relation. (A) The dependence of the relative error $\delta_{r_{J_{m}^{\prime }}}$ on the ratio of the number of clustering points (*n*_*cp*_) to the number of random points (*n*_*rp*_), *β* = *n*_*cp*_/*n*_*rp*_. The blue dashed line indicates a relative error of 5%. (B)The dependence of the relative error $\delta_{r_{J_{m}^{\prime }}}$ on the ratio of the density of clustering points (*ρ*_*c*_) to the density of random points (*ρ*_*r*_), *ρ*_*c*_/*ρ*_*r*_. The red dashed line indicates *ρ*_*c*_/*ρ*_*r*_ = 1.5; the green dot-dashed line indicates *ρ*_*c*_/*ρ*_*r*_ = 2; and the blue dotted line indicate an error of 10%.

“Noises” are almost always present in SMLM data, due to individual molecules not forming clusters, non-specific labeling, and/or false-positive localizations. A crucial question to examine is how this quantifier $r_{J_{m}^{\prime }}$ is affected by noises. As shown in Figs 3B and 4, $r_{J_{m}^{\prime }}$ is independent on the density of random noise points (or background points) in the data, strongly suggesting that it is likely to be robust to use $r_{J_{m}^{\prime }}$ to measure the clustering density of molecules (*ρ*_*c*_). To rigorously assess the robustness of the $r_{J_{m}^{\prime }}\;-\;\rho_{c}$ relation, we systematically investigated how $r_{J_{m}^{\prime }}$ deviates in the presence of various amount of noises for a given clustering density. First we looked at how $r_{J_{m}^{\prime }}$ changes with increasing ratios between the number of clustering points *n*_*cp*_ to the number of random (background) points *n*_*rp*_, *β* = *n*_*rp*_/*n*_*cp*_. We found that $r_{J_{m}^{\prime }}$ remained constant when there were up to ∼10 times more noise points than clustering points. The relative errors $\delta_{r_{J_{m}^{\prime }}}=\left|r_{J_{m}^{\prime }}-r_{J_{m}^{\prime }}^{*}\right|/r_{J_{m}^{\prime }}^{*}\times 100\%$ (where $r_{J_{m}^{\prime }}^{*}$ is without background points) were below 5% for *β* ≲ 10 (Fig 5A), indicating that the $r_{J_{m}^{\prime }}\;-\;\rho_{c}$ relation is very robust. In addition, as a more rigorous test, we also examined how the relative error $\delta_{r_{J_{m}^{\prime }}}$ behaves with increasing relative density between clusters and background, i.e., *ρ*_*c*_/*ρ*_*r*_. We found that $r_{J_{m}^{\prime }}$ was robust *ρ*_*c*_/*ρ*_*r*_ ≥ 2 with the relative error $\delta_{r_{J_{m}^{\prime }}}$ below 10% (Fig 5B). As *ρ*_*c*_/*ρ*_*r*_ decreased below 2, $\delta_{r_{J_{m}^{\prime }}}$ started to increase quickly, reaching ∼ 30 − 40% for *ρ*_*c*_/*ρ*_*r*_ = 1.5. Although not completely degraded, the accuracy of $r_{J_{m}^{\prime }}$ started to compromise for *ρ*_*c*_/*ρ*_*r*_ < 2. Therefore, it is suggested that $r_{J_{m}^{\prime }}$ be used for SMLM data with *ρ*_*c*_/*ρ*_*r*_ ≥ 2 to ensure the accuracy.

**Fig 6:**
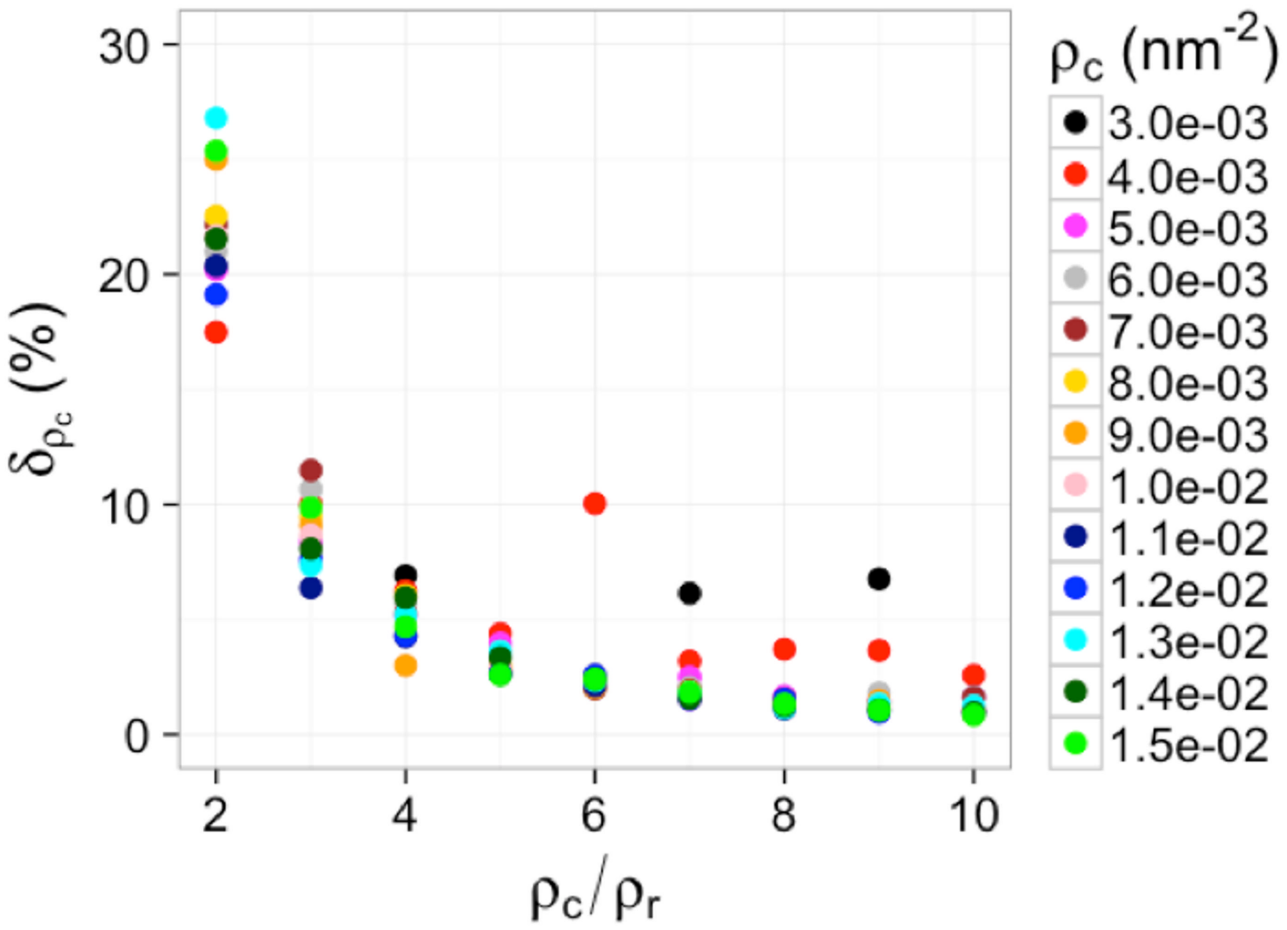
The dependence of the relative error *δ*_*ρ*_*c*__ on the ratio of the density of clustering points (*ρ*_*c*_) to the density of random points (*ρ*_*r*_), *ρ*_*c*_/*ρ*_*r*_, at various clustering densities.

It is expected that the error in measuring the density of clustering points is more relevant in real applications. Therefore, we also investigated the capability of using the $r_{J_{m}^{\prime }}\;-\;\rho_{c}$ “calibration” curve to estimate the clustering density of molecules in the presence of various amount of background noise points. Briefly, for each tested ground-truth clustering density (*ρ*_*c*_), we varied the density of background points (*ρ*_*r*_) such that *ρ*_*c*_/*ρ*_*r*_ ranged from 2 to 10. For each pair of (*ρ*_*c*_, *ρ*_*r*_), we generated 50 simulated data and computed *J*′(*r*) and $r_{J_{m}^{\prime }}$ for each simulation. The “measured” clustering density $\rho_{c}^{m}$ (averaged over the 50 simulations) was then obtained from the $r_{J_{m}^{\prime }}\;-\;\rho_{c}$ “calibration” curve, $\rho_{c}^{m}={\left(\left(r_{J_{m}^{\prime }}-b\right)/A\right)}^{-1/\alpha }$ (Fig 4A). The relative error in the measured clustering density was quantified by $\delta_{\rho_{c}}=\left|\rho_{c}^{m}-\rho_{c}\right|/\rho_{c}\times 100\%$. We observed that the error in “measured” clustering densities $\rho_{c}^{m}$ were close to the ground-truth density *ρ*_*c*_ (≲ 10% for *ρ*_*c*_/*ρ*_*r*_ ≥ 3 as shown in Fig 6) although the relative error increased as *ρ*_*c*_/*ρ*_*r*_ decreased (∼ 20 − 25% for *ρ*_*c*_/*ρ*_*r*_ = 2, shown in Fig 6), suggesting that it is robust to use $r_{J_{m}^{\prime }}$ to estimate clustering density (*ρ*_*c*_) in point patterns.

### *J*′(*r*) for heterogeneous clusters

**Fig 7:**
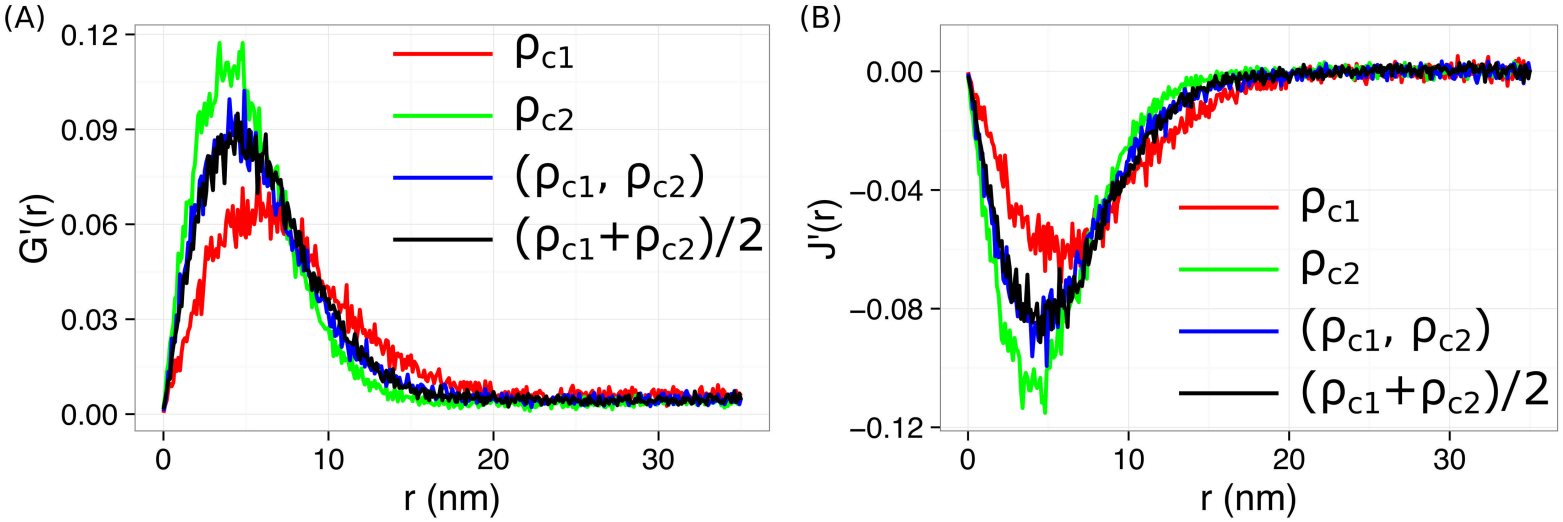
*G*′(*r*) and *J*′(*r*) for data with heterogeneous clusters with two different clustering densities.

It is known that, in certain applications, molecules of interest might form heterogeneous clusters [16, 23]. We examined heterogeneity arising from either clustering radius (*R*_*c*_) or clustering density (*ρ*_*c*_). Briefly, simulations were run for clusters with two different clustering radii (*R*_*c*1_ and *R*_*c*2_), or two different clustering densities (*ρ*_*c*1_ and *ρ*_*c*2_), in the presence of random noises. We noticed that $J_{\left(\rho_{c1},\rho_{c2}\right)}^{\prime }\left(r\right)$ from heterogeneous clusters with different clustering densities shifted both horizontally and vertically, and fell between the two curves from homogeneous clusters, $J_{\rho_{c1}}^{\prime }\left(r\right)$ and $J_{\rho_{c2}}^{\prime }\left(r\right)$ (Fig 7). In addition, we observed that $J_{\left(\rho_{c1},\rho_{c2}\right)}^{\prime }\left(r\right)$ overlapped very well with $J_{{\bar{\rho }}_{c}}^{\prime }\left(r\right)$ from a homogeneous sample with a clustering density equal to the algebraic mean, ${\bar{\rho }}_{c}=\left(\rho_{c1}+\rho_{c2}\right)/2$ (Fig 7). It is noted that *G*′(*r*) shows a similar behavior. Therefore, *G*′(*r*) and *J*′(*r*) report only the average clustering density throughout the region of interest; they cannot distinguish different clustering densities in heterogeneous clusters. In contrast, for heterogeneous clusters with different radii, $J_{\left(R_{c1},R_{c2}\right)}^{\prime }\left(r\right)$ shifted only in the vertical direction. The position of the valley, $r_{J_{m}^{\prime }}$, did not change for heterogeneous clusters with different radii (S4 Fig), which is expected because the $r_{J_{m}^{\prime }}\;-\;\rho_{c}$ relation does not depend on *R*_*c*_. In addition, we found that $J_{\left(R_{c1},R_{c2}\right)}^{\prime }\left(r\right)$ is equivalent to $J_{{\bar{R}}_{c}}^{\prime }\left(r\right)$ from homogeneous clusters with a radius of ${\bar{R}}_{c}=\sqrt{(R_{c1}^{2}+R_{c2}^{2})/2}$ (S4 Fig). Therefore, $r_{J_{m}^{\prime }}$ can be robustly used for heterogeneous clusters with different cluster sizes but the same clustering density.

### Application of *J*′(*r*) to *ptsG* mRNA in *E. coli* bacteria

**Fig 8:**
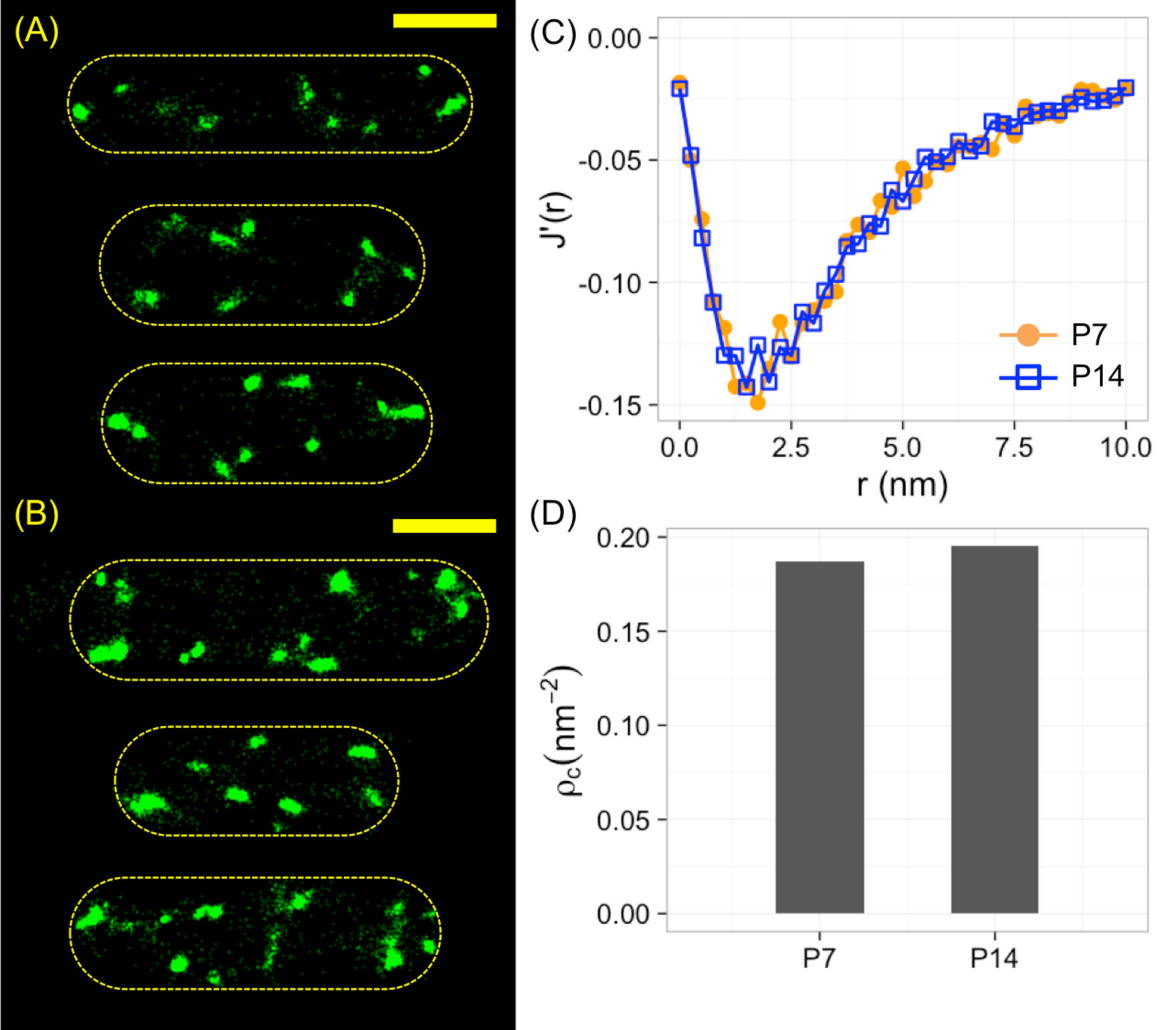
Application of *J*′(*r*) to *ptsG* mRNA in *E. coli* bacteria. (A, B) Super-resolved images of *ptsG* mRNA labeled through FISH by (A) 7 or (B) 14 fluorescent oligonucleotide probes. Scale bar = 1 *μ*m. (C) Computed *J*′(*r*) functions from (A) and (B). (D) Estimated clustering densities from (C).

As a simple example, we applied our method based on *J*′(*r*) and $r_{J_{m}^{\prime }}$ to measure the clustering density of *ptsG* mRNA, encoding a primary glucose transporter in *E. coli* bacteria. The *ptsG* mRNA were labeled through fluorescence *in situ* hybridization (FISH) by 7 Alexa 568-conjugated oligonucleotide probes, and were imaged by stochastic optical reconstruction microscopy (STORM) with a resolution of ∼ 20 nm in *x*/*y* and ∼ 50 nm in *z*. Three example bacteria were shown in Fig 8A. The average number of localizations per bacterial cell was 1576 ± 357 (mean ± standard error). The *J*′(*r*) function from the localizations were computed (orange curve in Fig 8C), which gave $r_{J_{m}^{\prime }}\approx 1.707$ nm and an estimated density of *ρ*_*c*_ ≈ 0.187 nm^−2^.

As a comparison, the same *ptsG* mRNA in *E. coli* bacteria were labeled by 14 probes via FISH, with three example cells shown in Fig 8B. The clusters of localizations appeared larger than those with 7 probes. Quantitatively, we measured 3090 ± 377 (mean ± standard error) localizations per bacterial cell, which was expected as the number of probes was doubled. However, as the spacing between 14 probes was similar to that between 7 probes, we expected that the density of localizations remained the same. We computed the *J*′(*r*) function for the sample labeled with 14 probes and found that the curve (blue curve in Fig 8C) overlapped well with that from the sample with 7 probes, indicating that the clustering density was unchanged. This observation was confirmed by examining $r_{J_{m}^{\prime }}$ (1.699 *vs.* 1.707) and the estimated clustering density (0.195 nm^−2^ *vs.* 0.187 nm^−2^, or ∼ 4% difference, Fig 8D), showing that the density estimated from $r_{J_{m}^{\prime }}$ was independent on the cluster size.

## Discussion

To conclude, we explored the possibility of utilizing nearest neighbor functions to quantify spatial patterns of molecules in single-molecule localization microscopy. We observed that the associated derivative functions, *G*′(*r*) and *J*′(*r*), can reliably report the clustering features of point patterns. We found that *J*′(*r*) is particularly useful because its position, $r_{J_{m}^{\prime }}$, relies exclusively on the density of clustering points (*ρ*_*c*_). More importantly, we showed that this $r_{J_{m}^{\prime }}\;-\;\rho_{c}$ relation is very robust in the presence of up to ∼10 times more noise points than clustering points, or when the relative density (*ρ*_*c*_/*ρ*_*r*_) is ≳ 2. As an example, we applied *J*′(*r*) and $r_{J_{m}^{\prime }}$ to robustly estimate the clustering of *ptsG* mRNA in *E. coli*.

In the current study, we chose not to exploit any border correction when computing the nearest neighbor functions. A simplest approach for border correction is the “reduced sample” method [41], which focuses on the points lying more than *r* away from the boundary of the region of interest. However, the “reduced sample” method discards much of the data, and therefore unacceptably wasteful. In addition, it’s particularly inappropriate in certain applications where points are preferentially located at the boundary, an example of which is the spatial organization of high-copy number plasmids in bacteria [14]. We note that more sophisticated methods for border correction are available, including the Kaplan-Meier correction [42] and the Hanisch correction [43], both are provided in the *spatstat* R-package [44, 45]. These edge corrections can be readily used in our method. However, for the sake of simplicity, uncorrected estimators for the nearest neighbor functions have been used in the current study.

We would like to emphasize that the current method based on nearest neighbor functions is nonparametric and robust. Computing the nearest neighbor functions and their derivatives does not require any parameters as human inputs, eliminating possible subjective biases that might exist in other algorithms such as DBSCAN and OPTICS. In addition, the performance of this method is robust in the presence of noise/background points. The nonparametric nature and robustness of the current method would allow broad applications in the field of single-molecule localization microscopy.

We expect several types of applications of our method in the field of SMLM. First, it can be used as a direct quantification of the clustering density (*ρ*_*c*_) of molecules in biological samples. Second, although it does not identify clusters by itself, our method, in combination with Ripley’s *H*′(*r*) function [32, 33], provides objective means to determine parameters (i.e., clustering density and cluster size) that can be used in other clustering-identification algorithm such as DBSCAN and Voronoï tessellation. In addition, in the current work, we focused on the $r_{J_{m}^{\prime }}-\rho_{c}$ relation for non-parametric measurement of the clustering density of molecules; however, we expect that it is possible to design ways to figure out other cluster features (such as *R*_*c*_ and *ρ*_*r*_) by taking advantage of the dependence of $G_{m}^{\prime }$ and $J_{m}^{\prime }$ on those features (S1 and S3 Figs), together with the information of *ρ*_*c*_.

## Methods

### Spherical contact distribution function *F*(*r*), nearest-neighbor distribution function *G*(*r*), and the *J* function *J*(*r*)

In a set of points, *X*, in the *k*-dimensional space, the spherical contact distribution function, or sometimes referred to as the empty space function, *F*(*r*), of *X* is defined as *F*(*r*) = *P*{*d*(*y*, *X*) ≤ *r*}, where *d*(*y*, *X*) = min{|*y* − *x*|: *x* ∈ *X*} is the distance from an arbitrary point, *y*, to the nearest point of the point process, *X* [34]. For a Poisson process with arrival intensity λ (equivalent to density in the context here) in the *k*-d space, $F_{p}\left(r\right)=1-\exp(-\lambda \frac{\pi^{k/2}r^{k}}{\Gamma (1+k/2)})$ [34]. The nearest-neighbor distribution function *G*(*r*) is very similar to *F*(*r*): *G*(*r*) = *P*^*y*^{*d*(*y*, *X*) ≤ *r*} where *P*^*y*^ is the Palm distribution, which is the conditional distribution of the entire process given that *y* is one point in *X* [34]. Therefore, *G*(*r*) is the distribution function of the distance from a point of the process to the nearest other point of the process, i.e., the “nearest-neighbor”. For a Poisson process in the *k*-d space, $G_{p}\left(r\right)=1-\exp(-\lambda \frac{\pi^{k/2}r^{k}}{\Gamma (1+k/2)})=F_{p}\left(r\right)$ [34]. In 1996, van Lieshout and Baddeley suggested using the quotient $J\left(r\right)=\frac{1-G(r)}{1-F(r)}$ to characterize a point process [35]. For a Poisson process, *J*_*p*_(*r*) = 1.

### Simulation and computation of *G*(*r*), *F*(*r*), *J*(*r*) and their derivatives

Sets of points were generated in R programing language [46]. In a region of interest with a width (*W*) and a height (*H*), *n*_*c*_ circular clusters with radii of *R*_*c*_ were randomly distributed. Each cluster contains random points at a density of *ρ*_*c*_. Poisson noise points were added randomly to the whole region of interest, with a density *ρ*_*r*_. The total number of clustering points ($n_{cp}=n_{c}\cdot \rho_{c}\cdot \pi R_{c}^{2}$) and the total number of noise points (*n*_*rp*_ = *ρ*_*r*_ ⋅ *WH*) define the noise level *β* = *n*_*rp*_/*n*_*cp*_.

Simulations were run using various sets of cluster features (*W*, *H*, *ρ*_*r*_, *ρ*_*c*_, *n*_*c*_, *R*_*c*_). For each set of features, 50–200 trials were run. The *G*(*r*), *F*(*r*), *J*(*r*) functions and their derivatives were computed using the *spatstat* package [44, 45], without applying any edge corrections.

### Bacterial sample preparation

Bacterial sample for imaging was prepared as previously published [13]. Briefly, an *E.coli* MG1655 derivative strain DJ480 (D. Jin, National Cancer Institute) was grown in MOPS EZ rich defined medium (TEKnova) supplemented with 0.2% fructose and 0.2% glucose at 37°C until OD600 reached 0.15-0.25. Cells were then fixed with 4% formaldehyde in 1X PBS and permeabilized with 70% ethanol. Chemically synthesized single molecule FISH (smFISH) probes (20 nucleotides each) were designed using Stellaris Probe Designer and ordered from Biosearch Technologies (http://www.biosearchtech.com). Seven or 14 probes against *ptsG* mRNA were then polled and labeled with Alexa Fluor 568 succinimidyl ester (Life Technologies). Permeabilized cells were washed once with FISH wash solution (10% formamide in 2X SSC) and resuspended in hybridization buffer (10% dextran sulfate and 10% formamide in 2X SSC) containing labeled FISH probes. Hybridization reactions were incubated in the dark at 30°C overnight. On the second day, the cells were washed three times with FISH wash solution. After the wash, the cells were pelleted, resuspended into 4X SSC. For imaging, cells were immobilized to poly-L-lysine treated 1.5 borosilicate chambered coverglass (Thermo Scientific^™^ Nunc^™^ Lab-Tek^™^).

### Super-resolution imaging and reconstruction

SMLM was performed on an inverted optical microscope (Nikon Ti-E with 100X NA 1.49 CFI HP TIRF oil immersion objective) with a yellow laser (561 nm, 150 mW, Coherent Obis LS) and a violet laser (405 nm, 25 mW, CrystaLaser) fiber coupled to the microscope body. Laser lines are reflected by a dichroic mirror (Chroma zt405/488/561/647/752rpc-UF3) having near-TIRF excitation. The emission signal was collected by the objective, filtered by emission filters (Chroma ET595/50m), and imaged on a 1024X1024 EMCCD camera (Andor iXon Ultra 888). Although a cylindrical lens with 10 m focal length (CVI RCX-25.4-50.8-5000.0-C-415-700) was inserted in the emission path, allowing 3D imaging [3], detected spots within a z slice (Δ*z* = ±100 nm) were used as a 2D projection. Violet laser power was modulated to keep the number of blinking-on spots above 50% of the number of cells in the field of view. When the number of blinking-on spots reached less than this, even with the maximum violet laser power, the acquisition was terminated. The power density lasers on the sample was ∼4300 *W* ⋅ *cm*^−2^ for yellow laser and the maximum power density for the violet laser was about 130 *W* ⋅ *cm*^−2^. Imaging buffer was composed of 10mM NaCl, 50mM Tris (pH = 8.0), 10% glucose, 30 Unit of glucose oxidase (G2133-10KU, Sigma-Aldrich) and 454.5 Unit of catalase (219001, EMD Millipore) in 4X SSC.

The data analysis algorithm was adopted from previous published [2, 3], and modified to handle multi-color and 3D images as previously published [13]. Briefly, all the pixels with intensity values greater than 3.5-4.5 fold of the standard deviation in each frame were identified. Within a 5-by-5 pixel area, local maximum intensity pixels whose intensity values were greater than its 24 surrounding pixels were found to represent the intensity peak of a single fluorophore. For identified peaks, a square region of 19×19 pixels surrounding local maximum intensity pixel was fitted with an Elliptical Gaussian function [3].

$$G\left(x,y\right)=h\times \exp\left(-2\frac{{\left(x-x_{0}\right)}^{2}}{w_{x}^{2}}-2\frac{{\left(y-y_{0}\right)}^{2}}{w_{y}^{2}}\right)+b$$

where *b* is the background level, *h* is the amplitude of the peak, *w*_*x*_ and *w*_*y*_ are elliptical widths, *x*_0_ and *y*_0_ are the center coordinates of the peak. The z-positions of the fluorophores were determined by comparing their *w*_*x*_ and *w*_*y*_ values to a calibration curve. Z-drift was prevented in real time Nikon perfect focus system. The horizontal drift was corrected during data analysis by fast Fourier transformation [13]. Finally, the acquired localization were used to generate reconstructed super-resolved images [3, 13, 14] and for quantitative analysis using *G*(*r*), *F*(*r*), and *J*(*r*), as well as their derivatives.

## Supporting information

S1 FigDependence of $G_{m}^{\prime }$ on the clustering features.(A) *ρ*_*c*_, (B) *ρ*_*r*_, (C) *R*_*c*_, (D) *N*_*c*_, (E) *W*, and (F) *H*.(TIF)

S2 FigDependence of $r_{G_{m}^{\prime }}$ on the clustering features.(A) *ρ*_*c*_, (B) *ρ*_*r*_, (C) *R*_*c*_, (D) *N*_*c*_, (E) *W*, and (F) *H*.(TIF)

S3 FigDependence of $J_{m}^{\prime }$ on the clustering features.(A) *ρ*_*c*_, (B) *ρ*_*r*_, (C) *R*_*c*_, (D) *N*_*c*_, (E) *W*, and (F) *H*.(TIF)

S4 Fig*G*′(*r*) and *J*′(*r*) from heterogeneous clusters with different radii.
${\bar{R}}_{c}=\sqrt{(R_{c1}^{2}+R_{c2}^{2})/2}$.(TIF)

## References

[1] Betzig E, Patterson GH, Sougrat R, Lindwasser OW, Olenych S, Bonifacino JS, et al Imaging intracellular fluorescent proteins at nanometer resolution. Science (New York, NY). 2006;313(5793):1642–1645. 10.1126/science.1127344 16902090

[2] Rust MJ, Bates M, Zhuang X. Sub-diffraction-limit imaging by stochastic optical reconstruction microscopy (STORM). Nature methods. 2006;3(10):793–795. 10.1038/nmeth929 16896339 PMC2700296

[3] Huang B, Wang W, Bates M, Zhuang X. Three-Dimensional Super-Resolution Reconstruction Microscopy. Health San Francisco. 2008;319(2):810–813. 10.1126/science.1153529 PMC2633023 18174397

[4] Heilemann M, Van De Linde S, Schüttpelz M, Kasper R, Seefeldt B, Mukherjee A, et al Subdiffraction-resolution fluorescence imaging with conventional fluorescent probes. Angewandte Chemie—International Edition. 2008;47(33):6172–6176. 10.1002/anie.200802376 18646237

[5] Klein T, Proppert S, Sauer M. Eight years of single-molecule localization microscopy. Histochemistry and Cell Biology. 2014;141(6):561–575. 10.1007/s00418-014-1184-3 24496595 PMC4544475

[6] Kopek BG, Shtengel G, Xu CS, Clayton DA, Hess HF. Correlative 3D superresolution fluorescence and electron microscopy reveal the relationship of mitochondrial nucleoids to membranes. Proceedings of the National Academy of Sciences. 2012;109(16):6136–6141. 10.1073/pnas.1121558109 PMC3341004 22474357

[7] Doksani Y, Wu JY, de Lange T, Zhuang X. Super-Resolution Fluorescence Imaging of Telomeres Reveals TRF2-Dependent T-loop Formation. Cell. 2013;155(2):345–356. 10.1016/j.cell.2013.09.048 24120135 PMC4062873

[8] Xu K, Zhong G, Zhuang X. Actin, spectrin, and associated proteins form a periodic cytoskeletal structure in axons. Science (New York, NY). 2013;339(6118):452–6. 10.1126/science.1232251 PMC3815867 23239625

[9] Ricci MA, Manzo C, García-Parajo MF, Lakadamyali M, Cosma MP. Chromatin Fibers Are Formed by Heterogeneous Groups of Nucleosomes In Vivo. Cell. 2015;160(6):1145–1158. 10.1016/j.cell.2015.01.054 25768910

[10] Stracy M, Lesterlin C, Garza de Leon F, Uphoff S, Zawadzki P, Kapanidis AN. Live-cell superresolution microscopy reveals the organization of RNA polymerase in the bacterial nucleoid. Proceedings of the National Academy of Sciences. 2015;112(32):E4390–E4399. 10.1073/pnas.1507592112 PMC4538611 26224838

[11] Haas BL, Matson JS, DiRita VJ, Biteen JS. Single-molecule tracking in live V ibrio cholerae reveals that ToxR recruits the membrane-bound virulence regulator TcpP to the toxT promoter. Molecular Microbiology. 2015;96(1):4–13. 10.1111/mmi.12834 25318589 PMC6025817

[12] Buss J, Coltharp C, Shtengel G, Yang X, Hess H, Xiao J. A Multi-layered Protein Network Stabilizes the Escherichia coli FtsZ-ring and Modulates Constriction Dynamics. PLOS Genetics. 2015;11(4):e1005128 10.1371/journal.pgen.1005128 25848771 PMC4388696

[13] Fei J, Singh D, Zhang Q, Park S, Balasubramanian D, Golding I, et al Determination of in vivo target search kinetics of regulatory noncoding RNA. Science. 2015;347(6228):1371–1374. 10.1126/science.1258849 25792329 PMC4410144

[14] Wang Y, Penkul P, Milstein JN. Quantitative localization microscopy combined with DNA smFISH reveals new features of the organization of high-copy number plasmids in bacteria. Biophys J. 2016;111(3):467–479. 27508432 10.1016/j.bpj.2016.06.033 PMC4982941

[15] Boettiger AN, Bintu B, Moffitt JR, Wang S, Beliveau BJ, Fudenberg G, et al Super-resolution imaging reveals distinct chromatin folding for different epigenetic states—nature16496.pdf. Nature. 2016;529(7586):418–422. 10.1038/nature16496 26760202 PMC4905822

[16] Sengupta P, Jovanovic-Talisman T, Skoko D, Renz M, Veatch SL, Lippincott-Schwartz J. Probing protein heterogeneity in the plasma membrane using PALM and pair correlation analysis. Nature Methods. 2011;8(11):969–975. 10.1038/nmeth.1704 21926998 PMC3400087

[17] Endesfelder U, Finan K, Holden SJ, Cook PR, Kapanidis AN, Heilemann M. Multiscale spatial organization of RNA polymerase in escherichia coli. Biophysical Journal. 2013;105(1):172–181. 10.1016/j.bpj.2013.05.048 23823236 PMC3699759

[18] Nan X, Collisson EA, Lewis S, Huang J, Tamgüney TM, Liphardt JT, et al Single-molecule superresolution imaging allows quantitative analysis of RAF multimer formation and signaling. Proceedings of the National Academy of Sciences of the United States of America. 2013;110(46):18519–24. 10.1073/pnas.1318188110 24158481 PMC3831949

[19] Caetano FA, Dirk BS, Tam JHK, Cavanagh PC, Goiko M, Ferguson SSG, et al MIiSR: Molecular Interactions in Super-Resolution Imaging Enables the Analysis of Protein Interactions, Dynamics and Formation of Multi-protein Structures. PLoS Computational Biology. 2015;11(12):1–30. 10.1371/journal.pcbi.1004634 PMC4676698 26657340

[20] Scarselli M, Annibale P, Radenovic A. Cell type-specific *β*2-adrenergic receptor clusters identified using photoactivated localization microscopy are not lipid raft related, but depend on actin cytoskeleton integrity. Journal of Biological Chemistry. 2012;287(20):16768–16780. 10.1074/jbc.M111.329912 22442147 PMC3351334

[21] Muranyi W, Malkusch S, Müller B, Heilemann M, Kräusslich HG. Super-Resolution Microscopy Reveals Specific Recruitment of HIV-1 Envelope Proteins to Viral Assembly Sites Dependent on the Envelope C-Terminal Tail. PLoS Pathogens. 2013;9(2). 10.1371/journal.ppat.1003198 23468635 PMC3585150

[22] Rubin-Delanchy P, Burn GL, Griffié J, Williamson DJ, Heard NA, Cope AP, et al Bayesian cluster identification in single-molecule localization microscopy data. Nature Methods. 2015;12(11). 10.1038/nmeth.3612 26436479

[23] Levet F, Hosy E, Kechkar A, Butler C, Beghin A, Choquet D, et al SR-Tesseler: a method to segment and quantify localization-based super-resolution microscopy data. Nature Methods. 2015;12(11). 10.1038/nmeth.3579 26344046

[24] Sengupta P, Lippincott-Schwartz J. Quantitative analysis of photoactivated localization microscopy (PALM) datasets using pair-correlation analysis. BioEssays. 2012;34(5):396–405. 10.1002/bies.201200022 22447653 PMC3659788

[25] Sengupta P, Jovanovic-Talisman T, Lippincott-Schwartz J. Quantifying spatial organization in point-localization superresolution images using pair correlation analysis. Nature protocols. 2013;8(2):345–54. 10.1038/nprot.2013.005 23348362 PMC3925398

[26] Ester M, Kriegel HP, Sander J, Xu X. A Density-Based Algorithm for Discovering Clusters in Large Spatial Databases with Noise. Second International Conference on Knowledge Discovery and Data Mining. 1996; p. 226–231.

[27] Daszykowski M, Walczak B, Massart DL. Looking for natural patterns in data. Part 1. Density-based approach. Chemometrics and Intelligent Laboratory Systems. 2001;56(2):83–92. 10.1016/S0169-7439(01)00111-3

[28] Ankerst M, Breunig MM, Kriegel Hp, Sander J. OPTICS: Ordering Points To Identify the Clustering Structure. SIGMOD’99 Proceedings of the 1999 ACM SIGMOD international conference on Management of data. 1999;28(2):49–60.

[29] Daszykowski M, Walczak B, Massart DL. Looking for natural patterns in analytical data. 2. Tracing local density with OPTICS. Journal of Chemical Information and Computer Sciences. 2002;42(3):500–507. 10.1021/ci010384s 12086507

[30] Deschout H, Shivanandan A, Annibale P, Scarselli M, Radenovic A. Progress in quantitative single-molecule localization microscopy. Histochemistry and Cell Biology. 2014;142(1):5–17. 10.1007/s00418-014-1217-y 24748502 PMC4072926

[31] Andronov L, Orlov I, Lutz Y, Vonesch JL, Klaholz BP. ClusterViSu, a method for clustering of protein complexes by Voronoi tessellation in super-resolution microscopy. Scientific reports. 2016;6(4):24084 10.1038/srep24084 27068792 PMC4828638

[32] Kiskowski MA, Hancock JF, Kenworthy AK. On the Use of Ripley’s K-Function and Its Derivatives to Analyze Domain Size. Biophysical Journal. 2009;97(4):1095–1103. 10.1016/j.bpj.2009.05.039 19686657 PMC2726315

[33] Lagache T, Lang G, Sauvonnet N, Olivo-Marin JC. Analysis of the Spatial Organization of Molecules with Robust Statistics. PLoS ONE. 2013;8(12):e80914 10.1371/journal.pone.0080914 24349021 PMC3857798

[34] Diggle PJ. Statistical Analysis of Spatial Point Patterns. 2nd ed London; New York: Hodder Education Publishers; 2003.

[35] van Lieshout MNM, Baddeley A. A non-parametric measure of spatial interaction in point patterns; 1996. Available from: http://wrap.warwick.ac.uk/18756/.

[36] Kerscher M, Pons-Borderia MJ, Schmalzing J, Trasarti-Battistoni R, Buchert T, Martinez VJ, et al A Global Descriptor of Spatial Pattern Interaction in the Galaxy Distribution. The Astrophysical Journal. 1999;513(2):543–548. 10.1086/306902

[37] Sherman E, Barr V, Manley S, Patterson G, Balagopalan L, Akpan I, et al Functional nanoscale organization of signaling molecules downstream of the T cell antigen receptor. Immunity. 2011;35(5):705–720. 10.1016/j.immuni.2011.10.004 22055681 PMC3225724

[38] Rossy J, Owen DM, Williamson DJ, Yang Z, Gaus K. Conformational states of the kinase Lck regulate clustering in early T cell signaling. Nature Immunology. 2012;14(1):82–89. 10.1038/ni.2488 23202272

[39] Garcia-Parajo MF, Cambi A, Torreno-Pina JA, Thompson N, Jacobson K. Nanoclustering as a dominant feature of plasma membrane organization. Journal of Cell Science. 2014;127(23):4995–5005. 10.1242/jcs.146340 25453114 PMC4260763

[40] Ehmann N, van de Linde S, Alon A, Ljaschenko D, Keung XZ, Holm T, et al Quantitative super-resolution imaging of Bruchpilot distinguishes active zone states. Nature communications. 2014;5:4650 10.1038/ncomms5650 25130366 PMC4143948

[41] Ripley BD. Statistical Inference for Spatial Processes. Cambridge: Cambridge University Press; 1988 Available from: http://ebooks.cambridge.org/ref/id/CBO9780511624131.

[42] Baddeley A, Gill RD. Kaplan-Meier estimators of distance distributions for spatial point processes. Annals of Statistics. 1997;25(1):263–292. 10.1214/aos/1034276629

[43] Hajstcsch KH. Some remarks on estimators of the distribution function of nearest neighbour distance in stationary spatial point processes. Series Statistics. 1984;15(3):409–412. 10.1080/02331888408801788

[44] Baddeley A, Turner R. spatstat: An R Package for Analyzing Spatial Point Patterns. Journal Of Statistical Software. 2005;12(6):1–42. 10.18637/jss.v012.i06

[45] Baddeley A, Rubak E, Turner R. Spatial Point Patterns: Methodology and Applications with {R}. London: Chapman and Hall/CRC Press; 2015 Available from: http://www.crcpress.com/Spatial-Point-Patterns-Methodology-and-Applications-with-R/Baddeley-Rubak-Turner/9781482210200/.

[46] R Core Team. R: A Language and Environment for Statistical Computing; 2016 Available from: https://www.r-project.org/.

