# Supplementary figures and images for: Robust nonparametric quantification of clustering density of molecules in single-molecule localization microscopy
Source: PLoS One. 2017 Jun 21;12(6):e0179975. doi: 10.1371/journal.pone.0179975 (PMC5479598; doi:10.1371/journal.pone.0179975)

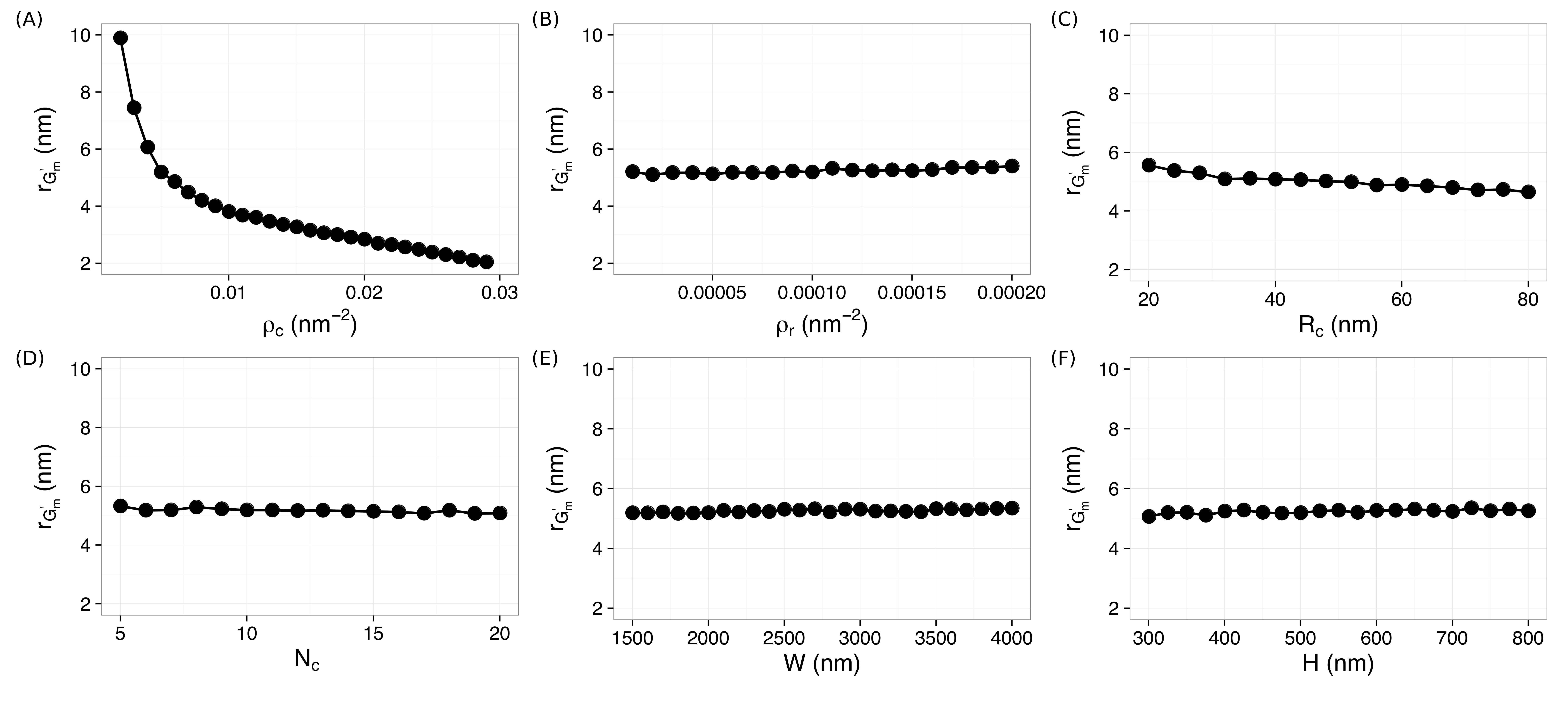

Supplement: S2 Fig — (A) ρc, (B) ρr, (C) Rc, (D) Nc, (E) W, and (F) H. (TIF) [file pone.0179975.s002.tif]

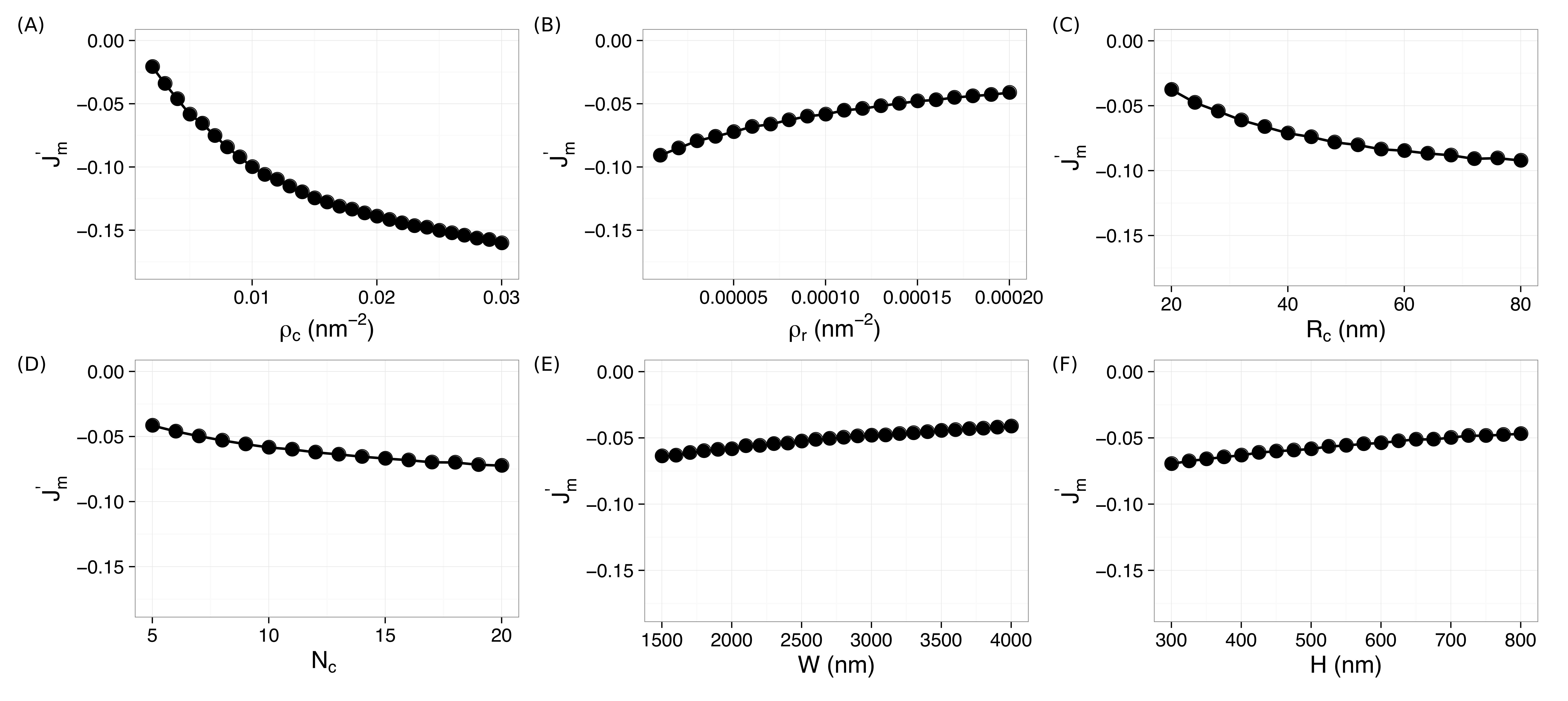

Supplement: S3 Fig — (A) ρc, (B) ρr, (C) Rc, (D) Nc, (E) W, and (F) H. (TIF) [file pone.0179975.s003.tif]

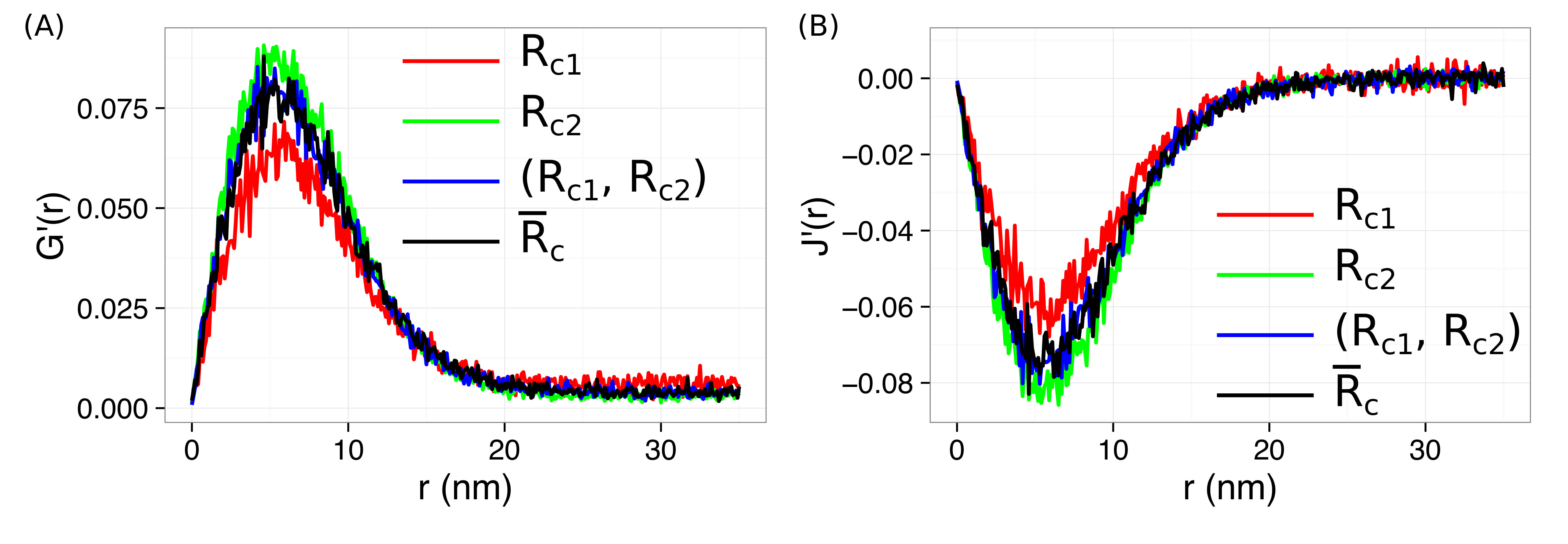

Supplement: S4 Fig — R¯c=(Rc12+Rc22)/2. (TIF) [file pone.0179975.s004.tif]
